# Supplementary material for: Benefits and limitations of three-dimensional printing technology for ecological research
Source: BMC Ecol. 2018 Sep 10;18:32. doi: 10.1186/s12898-018-0190-z (PMC6131837; doi:10.1186/s12898-018-0190-z)
Supplement: Supplementary file 1 — Additional file 1. Software for designing, modifying, and analyzing 3D files. [file 12898_2018_190_MOESM1_ESM.pdf]

Additional File 1 for:

Benefits And Limitations Of Three-Dimensional Printing Technology For Ecological Research

Jocelyn E. Behm[1,2*], Brenna R. Waite[1], S. Tonia Hsieh[3], and Matthew R. Helmus[1]

[1]Integrative Ecology Lab, Center for Biodiversity, Department of Biology, Temple University, Philadelphia, PA, USA

[2]Department of Ecological Science – Animal Ecology, VU University Amsterdam, Amsterdam, the Netherlands

[3]Department of Biology, Temple University, Philadelphia, PA, USA

***Correspondence:**
Jocelyn E. Behm
jebehm@temple.edu

Table 1: Software for designing, modifying, and analyzing 3D files

Name	Cost (in 2017)	Purpose	Edit 3D image	Generate image from photo	Build 3D image from scratch	Image analysis	Studies
3D Lightyear	Free	Preparing 3D file to print; inputs STL/SLC files and prepares them for part building in other software	Yes	No	No	No	[1]
Agisoft PhotoScan	Reduced rate academic licenses	Photogrammetry software for processing digital images and generating 3D spatial data	Yes	Yes	No	Yes	[2]
Amira	Contact sales rep	Visualizing, manipulating, and understanding data from CT, MRI, microscopy, and	No	Yes	No	Yes	[3]

		other imaging methods.					
AutoCAD (Autodesk)	Reduced rate academic licenses	Computer-aided design and drafting software for 3D modelling	Yes	No	No	No	[4]
Blender	Free	3D image modeling, rigging, rendering	Yes	No	Yes	No	[5]
Checkpoint	Reduced rate academic licenses	3D modeling, landmark collection and editing.	Yes	Yes	No	Yes	[6]
CTan	Free	2D and 3D micro-CT dataset analysis and visualization	No	No	No	Yes	[7]
FreeCAD	Free	3D modeling and modifying	Yes	No	Yes	No	[8]
Geomorph	Free	Geometric morphometric shape analysis in R environment	Yes	No	No	Yes	-
ImageJ	Free	Image enhancement, analysis, and editing	Yes	No	No	Yes	[5,7]
Inventor (Autodesk)	Free Academic License	3D mechanical design, simulation, tooling	Yes	No	No	No	[5]
InVesalius	Free	Reconstruction of CT & MRI	No	No	No	Yes	[7]
Maya (Autodesk)	Free Academic License	3D animation, modeling, rendering, simulation	Yes	No	Yes	No	This study
MeshLab	Free	Editing, cleaning, healing, inspecting, rendering and	Yes	No	No	No	[7]

		converting unstructured 3D scans					
Meshmixer (Autodesk)	Free	3D sculpting and image manipulation	Yes	No	Yes	Yes	[9]
MorphoJ	Free	Quantitative analysis of geometric morphometrics	No	No	No	Yes	[10,11]
OpenSCAD	Free	Focuses on CAD rather than artistic aspects of generating and manipulating 3D modelling	Yes	Yes	Yes	No	[12]
PhotoModeler Scanner	$2,495	Alternative to 3D laser scanning to create 3D models from photographs	No	Yes	No	Yes	[10]
PhyloNimbus	Free	Landmarks & linear/curve measurement of 2D or 3D projects	No	No	No	Yes	-
Polyworks (Innovmetric)	Contact sales rep	Comprehensive 3D modeling software system with universal file formats	Yes	Yes	Yes	Yes	-
SketchUp (Google)	Reduced rate academic licenses	Designing 3D imagery from scratch	No	No	Yes	No	[13]
Solid Works	Contact sales rep	Most aspects of 3D image generation and manipulation	Yes	Yes	Yes	Yes	[12]
TinkerCad	Free	Highly accessible, web-based tool for designing new 3D models and, modifying existing 3D models	Yes	No	Yes	No	-

Literature Cited

1. Cvetkovic C, Raman R, Chan V, Williams BJ, Tolish M, Bajaj P, et al. Three-dimensionally printed biological machines powered by skeletal muscle. PNAS. 2014;111:10125–30.

2. Marchal AFJ, Lejeune P, de Bruyn PJN. Virtual plaster cast: digital 3D modelling of lion paws and tracks using close-range photogrammetry. J. Zool. 2016;300:111–9.

3. Vanderelst D, Peremans H, Razak NA, Verstraelen E, Dimitriadis G. The aerodynamic cost of head morphology in bats: Maybe not as bad as it seems. PLOS ONE. 2015;10:e0118545.

4. Rochman D, Luna ED. Prototyping the complex biological form of the beetle *Deltochilum lobipes* via 2D geometric morphometrics landmarks and descriptive geometry for 3D printing. Computer-Aided Design and Applications. 2017;14:107–16.

5. Igic B, Nunez V, Voss HU, Croston R, Aidala Z, López AV, et al. Using 3D printed eggs to examine the egg-rejection behaviour of wild birds. PeerJ. 2015;3:e965.

6. Ledoux L, Boudadi-Maligne M. The contribution of geometric morphometric analysis to prehistoric ichnology: the example of large canid tracks and their implication for the debate concerning wolf domestication. Journal of Archaeological Science. 2015;61:25–35.

7. Dal Ferro N, Morari F. From real soils to 3D-printed soils: Reproduction of complex pore network at the real size in a silty-loam soil. Soil Science Society of America Journal. 2015;79:1008–17.

8. Watson CM, Francis GR. Three dimensional printing as an effective method of producing anatomically accurate models for studies in thermal ecology. Journal of Thermal Biology. 2015;51:42–6.

9. Gutierrez-Heredia L, Benzoni F, Murphy E, Reynaud EG. End to End Digitisation and Analysis of Three-Dimensional Coral Models, from Communities to Corallites. PLOS ONE. 2016;11:e0149641.

10. Chiari Y, Wang B, Rushmeier H, Caccone A. Using Digital Images to Reconstruct Three-Dimensional Biological Forms: A New Tool for Morphological Studies. Biological Journal of the Linnean Society. 2008;95:425–36.

11. Lamb T, Pollard R, Bond JE. Genetic variation corroborates subspecific delimitation in the Namib fog-basking beetle, Onymacris unguicularis (Haag) (Tenebrionidae, Coleoptera). Zookeys. 2013;47–60.

12. Berry D, Selby RD, Horvath JC, Cameron RH, Porqueras D, Stouthamer R. A modular system of 3D printed emergence traps for studying the biology of shot hole borers and other scolytinae. J Econ Entomol. 2016;109:969–72.

13. Chetverikov PE. Hidden diversity of endoparasitic eriophyoid mites: two new Novophytoptus Roivainen, 1947 (Acari: Eriophyoidea: Phytoptidae) species from the parenchymatous tissues of rushes (Juncaceae). Zootaxa. 2015;4006:481–505.
