# Supplementary material for: Benefits and limitations of three-dimensional printing technology for ecological research
Source: BMC Ecol. 2018 Sep 10;18:32. doi: 10.1186/s12898-018-0190-z (PMC6131837; doi:10.1186/s12898-018-0190-z)
Supplement: Supplementary file 2 — Additional file 2. Online libraries of 3D imagery relevant for ecological research (as of 2017). [file 12898_2018_190_MOESM2_ESM.pdf]

Additional File 2 for:

Benefits And Limitations Of Three-Dimensional Printing Technology For Ecological Research

Jocelyn E. Behm[1,2*], Brenna R. Waite[1], S. Tonia Hsieh[3], and Matthew R. Helmus[1]

[1]Integrative Ecology Lab, Center for Biodiversity, Department of Biology, Temple University, Philadelphia, PA, USA

[2]Department of Ecological Science – Animal Ecology, VU University Amsterdam, Amsterdam, the Netherlands

[3]Department of Biology, Temple University, Philadelphia, PA, USA

***Correspondence:**
Jocelyn E. Behm
jebehm@temple.edu

Table 2: Online libraries of 3D imagery relevant for ecological research (as of 2017)

Name	Website	Description
Cgtrader	https://www.cgtrader.com/3d-models	Repository of professional 3D models
GrabCAD	https://grabcad.com/	Allows for printing straight from CAD; has a collaboration tool for working on group projects; also has library of free 3D imagery files
NIH 3D Print Exchange	https://3dprint.nih.gov/	Provides scientifically accurate and medically applicable 3D models that are readily compatible with 3D printers.
Sketchfab	https://sketchfab.com/	Platform for sharing 3D images on social media; also allows for 3D content to be embedded on webpages and social media
Thingiverse (by Makerbot)	http://www.thingiverse.com/	Largest online community for sharing 3D models on an open platform
Dryad	http://datadryad.org/	Repository for scientific files including 3D media
STL Finder	https://www.stlfinder.com/	Search engine for 3D models
